# Orthopædics: A Systematic Treatise upon the Prevention and Correction of Deformities

**Published:** 1867-03

**Authors:** 


ORTHOPÆDICS: A SYSTEMATIC TREATISE UPON THE PREVENTION AND CORRECTION OF DEFORMITIES. By DAVID PRINCE, M.D. Published by LINDSAY & BLAKISTON, Philadelphia.

This work of a well-known surgeon of Jacksonville, Ill., comes to us to supply an urgent want in our surgical literature. Our ordinary surgical text-books are wholly insufficient to guide the general practitioner in orthopædic matters, and even the special works on this subject are mostly of a crude and unsatisfactory sort, though containing many valuable ideas. Dr. PRINCE'S work has the merit of containing a careful résumé of the best ideas of the best men, combined with his own special improvements. He has tried to so systematize and simplify the complexities of the subject as to lay the whole of it plainly before the general practitioner. In pursuance of this object, he has taken especial pains to devise forms of apparatus which can be made *extempore*, by members of the profession who have no access to regular instrument makers—a plan which, if carried out, will very much widen the usefulness of orthopædic surgery.

The wôrk is an octavo of 240 pages, and illustrated by nearly 100 wood-cuts.

Part First, consists of a concise discussion of the principles of the science, including the pathology of deformities and of the various joint-diseases, and shows the measures and mechanical appliances adapted to remedy them. It cannot be said that Dr. PRINCE has given an exhaustive view of the pathology of these complaints. Those who wish this must look to other writers. But he has done what to the general practitioner is far more useful, he has given the *main principles* of the pathology, in close connection with the principles of the treatment.

Part Second, deals more closely with special diseases. He commences with hip-disease, because it is, as he remarks, "the lion of its class." After describing the disease, he shows that the suggestion of the great modern principle of extension for inflamed joints was first made by Brodie; that Dr. March, of Albany, came very near producing the first portable splint for this purpose, but that Dr. H. G. Davis, of New York, first put the principle into a compact and manageable form, and really made the first good hip-disease splint. After figuring and describing the various splints now in use, Dr. Prince describes one of his own, which may be constructed by any physician, with the help of a common blacksmith, and which is equally applicable to inflammations of the hip and of the knee-joints.

After treating fully of hip-disease and of inflammations of the knee, the author remarks that, inflammations of the ankle "hardly admit of extension." In this we differ from him. Extension can be applied effectually to the ankle, but it is more difficult, and requires more attention from the surgeon than that of the hip or knee.

The next 50 pages is devoted to spinal curvatures, which are treated of in a sound and practical manner, and two forms of apparatus are introduced, of the author's invention, which can be made by any ordinary blacksmith, thus placing their construction within the reach of the remotest country practitioner. Several illustrations and descriptions of other appliances are also given. It is to be hoped that this plan of devising home-made apparatus will not only greatly benefit many patients who are too poor to go abroad for assistance, but will also turn the attention of general practitioners to this subject, and lead them to comprehend the fact that no ready-made apparatus can be bought, for spinal diseases, which will be of the least use to the patient. Everything must be made to fit the case in hand, or it will be of no use whatever. The so-called spinal supporters, kept ready-made in the instrument stores, are the most worthless trash ever offered to the public. The remainder of the work is occupied with talipes and other deformities. The author presents several excellent inventions of his own, besides a careful condensation of the opinions of others.

On the whole, this work of Dr. Prince, though it might be improved in some respects, is, perhaps, the most practically useful treatise upon orthopædics which has appeared in this country, and we cheerfully commend it to the profession.

